# Doping Tunable CDW Phase Transition in Bulk 1T-ZrSe_2_

**DOI:** 10.1021/acs.nanolett.4c06377

**Published:** 2025-01-15

**Authors:** Andreas Ørsted, Alessandro Scarfato, Céline Barreteau, Enrico Giannini, Christoph Renner

**Affiliations:** Department of Quantum Matter Physics, University of Geneva, 24, Quai Ernest-Ansermet, 1211 Geneva 4, Switzerland

**Keywords:** Scanning Tunneling Microscopy, Scanning Tunneling Spectroscopy, Tunability, Charge Density Wave

## Abstract

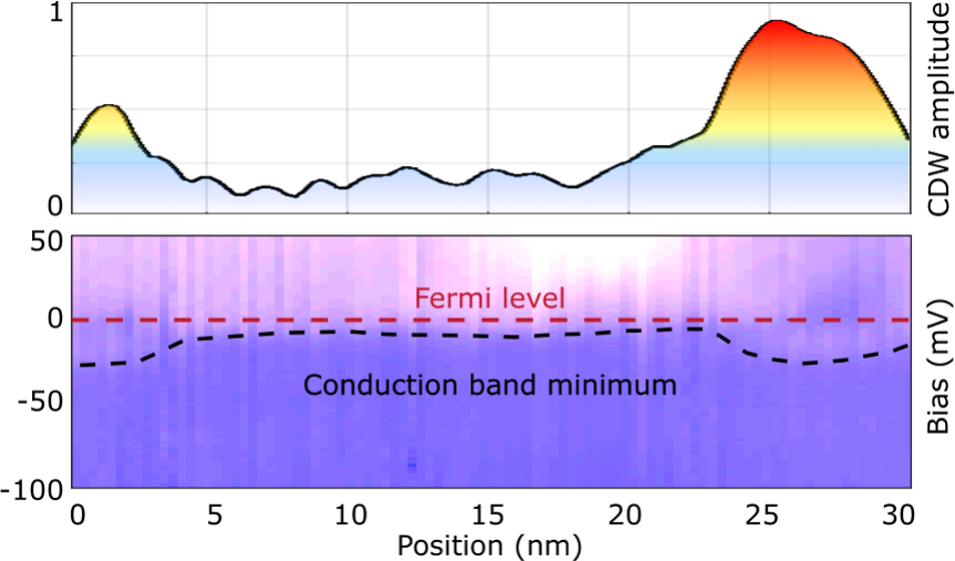

Tunable electronic properties in transition metal dichalcogenides (TMDs) are essential to further their use in device applications. Here, we present a comprehensive scanning tunneling microscopy and spectroscopy study of a doping-induced charge density wave (CDW) in semiconducting bulk 1T-ZrSe_2_. We find that atomic impurities that locally shift the Fermi level (*E*_*F*_) into the conduction band trigger a CDW reconstruction concomitantly to the opening of a gap at *E*_*F*_. Our findings shed new light on earlier photoemission spectroscopy and theoretical studies of bulk 1T-ZrSe_2_ and provide local insight into the electron-doping-mediated CDW transition observed in semiconducting TMDs.

Since the discovery of graphene, extensive theoretical and experimental efforts have been devoted to two-dimensional (2D) and quasi-2D systems. The investigations into their intriguing physical properties have generated numerous highly influential contributions to the field of material physics, including 2D superconductivity,^1^ heterostructure devices,^2^ moiré physics,^3^ topology,^4^ spin and charge density waves,^5^ and many more. However, even though our understanding of these systems has grown massively in recent years with both theoretical and experimental advances, individual pieces of the puzzle remain undiscovered or elusive. Here, we focus on the charge density wave (CDW) phase in a quasi-2D system, which is of particular interest because of the many open questions concerning its formation mechanism and its interplay with other electronic phases such as superconductivity.^6−8^

The CDW formation in one dimension is well-established: a metallic chain of atoms distorts into a gapped state concomitant with a modulated charge distribution due to Fermi surface nesting (FSN). In 2D and quasi-2D compounds, in addition to FSN,^9−12^ other possible mechanisms are proposed, the most common being Jahn–Teller-like electron–phonon coupling.^13−19^

We use scanning tunneling microscopy (STM) and spectroscopy (STS) in ultrahigh vacuum to investigate in situ cleaved surfaces of bulk 1T-ZrSe_2_ (hereafter ZrSe_2_) at 4.4 K. Depending on scanning bias voltage, we find dispersing and nondispersing periodic charge modulations. At small negative tunneling biases, we observe nondispersing charge modulations akin to the 2*a* × 2*a* modulation observed at the semiconductor-to-metal transition in few-layer thin ZrSe_2_ flakes grown by molecular beam epitaxy on graphitized SiC(0001).^20^ A well-defined nondispersing ***q***-vector and contrast inversion across the CDW gap at the Fermi level (*E*_*F*_) underline the CDW nature of this modulation.^21^ At a tunneling bias of +100 meV or higher above *E*_*F*_, we find dispersive charge modulations with a wavelength that increases with energy. These modulations are clearly quasiparticle interference (QPI) patterns incompatible with a CDW. They are associated with a large density of state (DOS) in the conduction band and observed only above *E*_*F*_.

The scanning probe data discussed here provide compelling evidence that the onset of the CDW phase transition in bulk ZrSe_2_ is associated with a shift of the Fermi level into the conduction band due to local electron doping by impurities. A doping-induced CDW concomitant to a semiconductor-to-metal phase transition has previously been reported in few-layer thin ZrSe_2_ grown on graphene.^20^ However, in contrast to these previous experiments, we can unambiguously attribute the CDW’s origin to doping, excluding any measurable strain or reduced dimensionality. Our data reveal a remarkable correlation between the shift of the Fermi level into the conduction band and the appearance of a CDW with the opening of a gap at *E*_*F*_. ZrSe_2_ is the second system after potassium-doped MoS_2_ where a doping-dependent CDW reconstruction is observed,^22^ demonstrating doping as a possible tuning parameter of the CDW ground state in semiconductor TMD systems. Carrier doping in ZrSe_2_ and the resulting shift of the Fermi level are likely due to native defects in the as-grown crystals. These defects have been identified as Se vacancies,^23,24^ Zr intercalations,^25^ or more complex atomic disorder involving both Se vacancies and Zr interstitials,^26^ all acting as electron donors, and they depend on the processing conditions. Small local deviations from stoichiometry during the growth process may result in large concentrations of such defects and even the formation of parent phases ZrSe_3_ and Ze_3_Se_4_. It is, therefore, important to specify the processing conditions in order to control and reproduce the sample quality.

Crystals of ZrSe_2_ were grown by the chemical vapor transport (CVT) method, but, despite being traditionally grown using iodine, we employed ZrCl_4_ as a transport agent. The use of transition metal chloride instead of pure halides has proven to be successful for growing high-quality crystals of many TMD materials.^27,28^ A 99.9% pure ZrCl_4_ powder was added to 99.9% pure Zr lumps and 99.999% Se shots, according to the reaction equation 0.9Zr + 0.1ZrCl_4_ + 2Se → ZrSe_2_ + 0.2Cl_2_, and sealed under vacuum inside a quartz tube (inner diameter 8 mm, length ≃120 mm). A total mass of 0.3 g was introduced in each ampule. The ampule was placed horizontally in a tubular furnace and annealed in a temperature gradient (*T*_*hot*_ = 910 °C, *T*_*cold*_ = 830 °C) for 120 h, then quickly pulled out to room temperature. Gray-greenish crystalline platelets with a metallic luster were extracted from the cold end of the reactor and confirmed to be [00l]-oriented ZrSe_2_ 2D crystals. The chemical analysis, performed by energy-dispersive X-ray spectroscopy in a scanning electron microscope, revealed a little excess of Zr, with a composition varying from ZrSe_2_ to Zr_1.15_Se_1.85_, in agreement with previous reports. The equilibrium phase diagram of Zr–Se has not been assessed; however in analogy to the similar systems Ti–Se, Zr–S, and Zr–Te, such a finite composition range is expected, which is at the origin of the native defects that tune the electronic properties investigated here. The crystals were cleaved in situ shortly before inserting into the STM head.

We carried out STM and STS measurements using etched tungsten tips in a SPECS JT-STM setup operated at ≈4.4 K at a base pressure around 4 × 10^–11^ mbar. STM measurements were performed in constant current mode, with a bias voltage applied to the sample. We have added small windowing to smooth the edges of all images, resulting in cleaner Fourier transforms. STS conductance maps were acquired using a standard lock-in technique with 5 mV AC modulation. Current imaging tunneling spectroscopy (CITS) maps^29^ were taken on a 170 × 170 grid in a 30 × 30 nm^2^ window. Each spectrum consists of 183 equally spaced points between −300 and +50 mV and is low-pass filtered using a moving average over five points. The CITS maps are normalized by dividing the *dI*/*dV*(*V*) signal by (*I*/*V*). The Fourier components were isolated using soft 21-by-21-pixel Gaussian windows centered on the Fourier peaks.

In Figure 1, we present three constant-current STM topography images acquired at different biases over the same area of an in situ cleaved ZrSe_2_ surface at 4.4 K. Positive bias images reveal a very sharp atomic resolution with different atomic impurity features (Figure 1a). Reducing the bias to small negative voltages (Figure 1b), we still resolve the atomic lattice, albeit less sharply, but we no longer see most of the atomic defects resolved at a positive bias. The most remarkable aspect of small negative bias images is the appearance of static 2*a* charge modulations aligned with the atomic lattice, where *a* is the atomic lattice constant. These modulations are only resolved for bias voltages between −800 mV and +50 mV. At larger negative bias (<−900 mV), STM images reveal nm-sized bright and dark spots on a homogeneous background, with no atomic-scale resolution (Figure 1c). We associate these spots with the charge inhomogeneities observed at a small negative bias in Figure 1b, but with opposite contrast. This change, in contrast, is a direct consequence of the semiconducting gap,^30^ as explained below.

**Figure 1:**
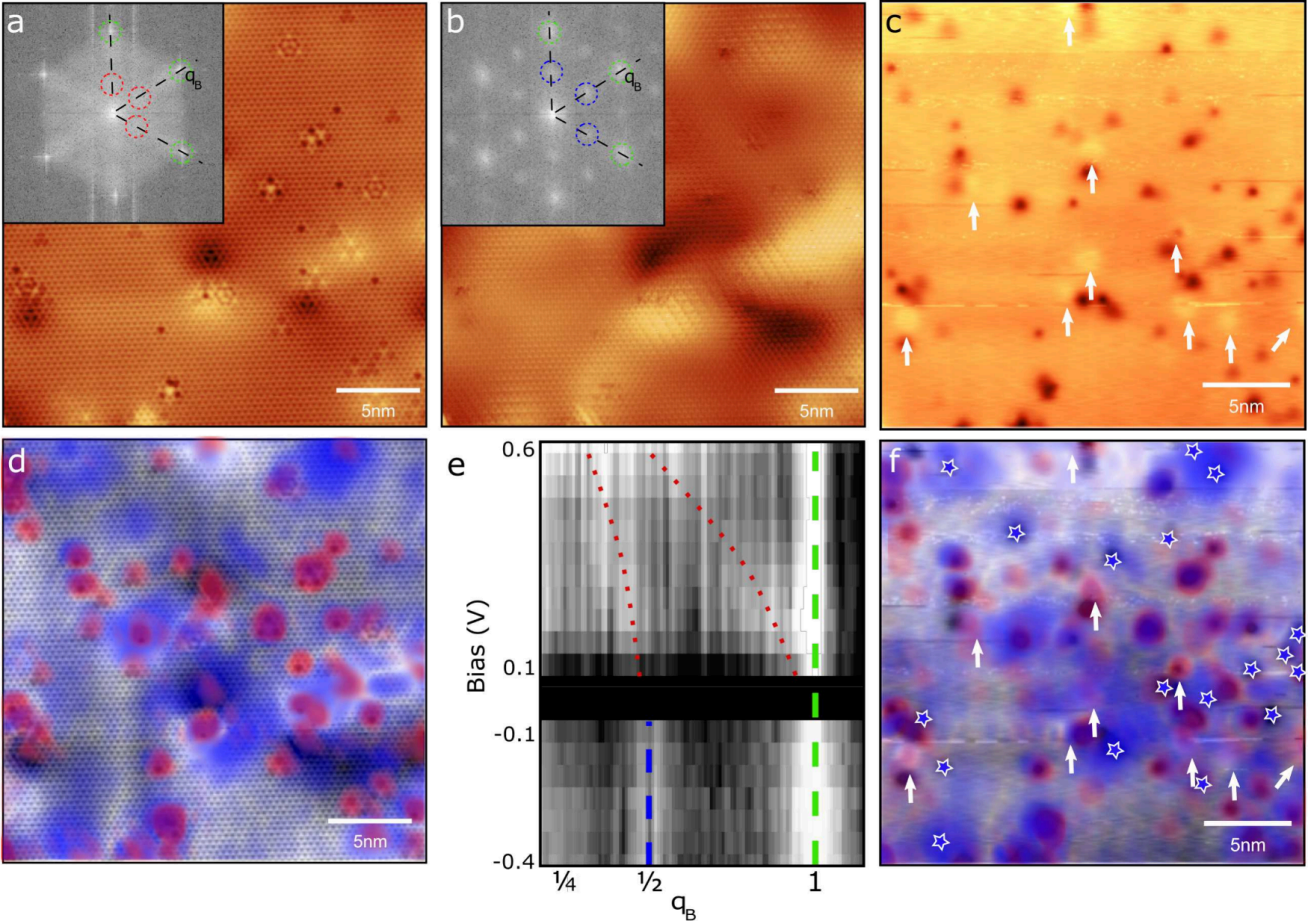
30 × 30 nm^2^ STM topography of the same region of ZrSe_2_ measured at (a) +400 mV, (b) −400 mV, and (c) −1.1 V bias. The insets show the corresponding Fourier transforms. (d) Topography in (a) with overlaid inverse Fourier transforms restricted to the blue and red areas of the FTs in (a) and (b). (e) Trace from the Γ-point to the Bragg peaks as a function of energy averaged along the dashed lines in the FTs (no data are available near *E*_*F*_). The green dashed line marks the Bragg peak, the blue one marks the CDW peak, and the red dotted lines mark two dispersing QPI features. (f) Topography in (c) with overlaid inverse Fourier transforms restricted to the blue and red areas of the FTs in (a) and (b). The white arrows indicate selected hole-doping defects, and the blue stars indicate the electron-doping defects visible only at high negative bias.

The periodic modulations observed in the topographic and spectroscopic images can be easily isolated by Fourier transform (FT), with distinct features observed in the positive and negative bias images of ZrSe_2_. The crisp atomic lattice resolved at positive bias yields well-defined Bragg peaks at *q*_*B*_ outlined in green in the inset of Figure 1a. At negative bias, the Bragg peaks are weaker and significantly more diffuse (inset of Figure 1b). The focus of this study is on the Fourier components near  present at both polarities but with very different bias dependencies (Figure 1e). FTs of positive bias images show very weak peaks near *q*_1/2_ outlined in red in Figure 1a. Their center of mass shifts toward smaller ***q***-values with increasing imaging voltage (Figure 1e) while becoming more and more diffuse. Higher positive bias images show finite scattering amplitude in the entire hexagonal region defined by the six Bragg peaks. In contrast, the FTs of negative bias images reveal more defined peaks at *q*_1/2_ outlined in blue in Figure 1b. These peaks do not shift as a function of tunneling bias (Figure 1e), and no additional diffuse amplitude covers the hexagonal region defined by the Bragg peaks.

The spatial distribution and amplitude of the dispersing and nondispersing modulations introduced above can be obtained through the inverse FT of the components outlined in blue and in red in Figure 1a,b. The composite images obtained by overlaying these spatial distributions on the topographic images in Figure 1d,f clearly show that the dispersing (red) and nondispersing (blue) components originate in different regions on the surface. The dispersing modulations develop in the immediate vicinity of the sharp atomic defects observed in positive bias topographic images and rapidly decay with distance from them. In contrast, the nondispersing components are primarily located in between them. As demonstrated below, the dispersing charge modulations observed above +100 mV are the result of quasiparticle interference, whereas the nondispersing modulations with periodicity 2*a* observed between −800 and +50 mV are CDWs. The latter appear only in regions where local electron doping shifts the Fermi level into the conduction band.

The periodic modulations observed near *q*_1/2_ at positive and negative biases are of a very different nature. The dispersing ***q***-vector as a function of tunneling bias (Figure 1e) and the absence of contrast inversion clearly identify the modulations observed at positive bias above +100 mV as quasiparticle interference. They are associated with the sharp rise of the tunneling conductance with bias above the semiconducting gap, corresponding to a large density of states available for scattering. Many bands contribute to this DOS, with many possible scattering vectors which ultimately cover the entire phase space inside the hexagon defined by the Bragg peaks (see inset in Figure 1a). Note that the Bragg peaks are very sharp, indicating that they are not affected by higher-order components that would be expected if the peaks near *q*_1/2_ were a CDW. On the other hand, the periodic modulations observed between −800 mV and +50 mV correspond to well-defined ***q***-vectors (Figure 1e) and comply with all the criteria expected for a CDW: their ***q***-vector of  does not depend on the imaging voltage, they appear alongside a gap in the local DOS at the Fermi level, and their contrast is inverting across this gap in topographic and spectroscopic images.^21^ The broad *q*_1/2_ components reflect the lack of long-range order and the limited size of the CDW domains. Here, the Bragg peaks are similarly diffuse, consistent with a commensurate CDW at *q*_1/2_ whose higher-order components will coincide with and affect the peaks at *q*_*B*_.

As seen in the conductance map in Figure 2a, the CDW revealed here by STM in bulk ZrSe_2_ is a 2*a* × 2*a* charge modulation with different strengths of its three characteristic ***q***-vectors, giving it a 1Q, 2Q, or 3Q character^31^ depending on location on the surface. The stripy character of the CDW in some regions has been reported previously on other TMD compounds. The associated suppression of some of the ***q***-vectors has been explained in terms of strain^32−34^ or local doping.^35^ Some dopant atoms may induce local strain (Figure S4), as evidenced by the lattice parameter dependence on the Se/Zr ratio^23^ and supported by theoretical predictions.^36^ While extended regions of minor strain, potentially altering electron–phonon coupling, cannot be ruled out, we observe lower electron doping in the 1Q regions compared to the 3Q regions. Additionally, strain is negligible in CDW regions with the highest modulation amplitude, suggesting local doping rather than strain as the primary driver of CDW formation. We propose that doping in the 1Q region is insufficient to fully transition to a 3Q state with local fluctuations, possibly aided by minor strain, favoring one or two components. On the other hand, we find that the conduction band minimum is closer to *E*_*F*_ in these regions, indicating a lower degree of electron doping compared to the 3Q regions. The CDW contrast inversion across the gap near *E*_*F*_ is seen in Figure 2b, where we plot the conductance averaged along *Y* as a function of position *X* and energy in the 1Q region outlined by the white box in Figure 2a.

**Figure 2:**
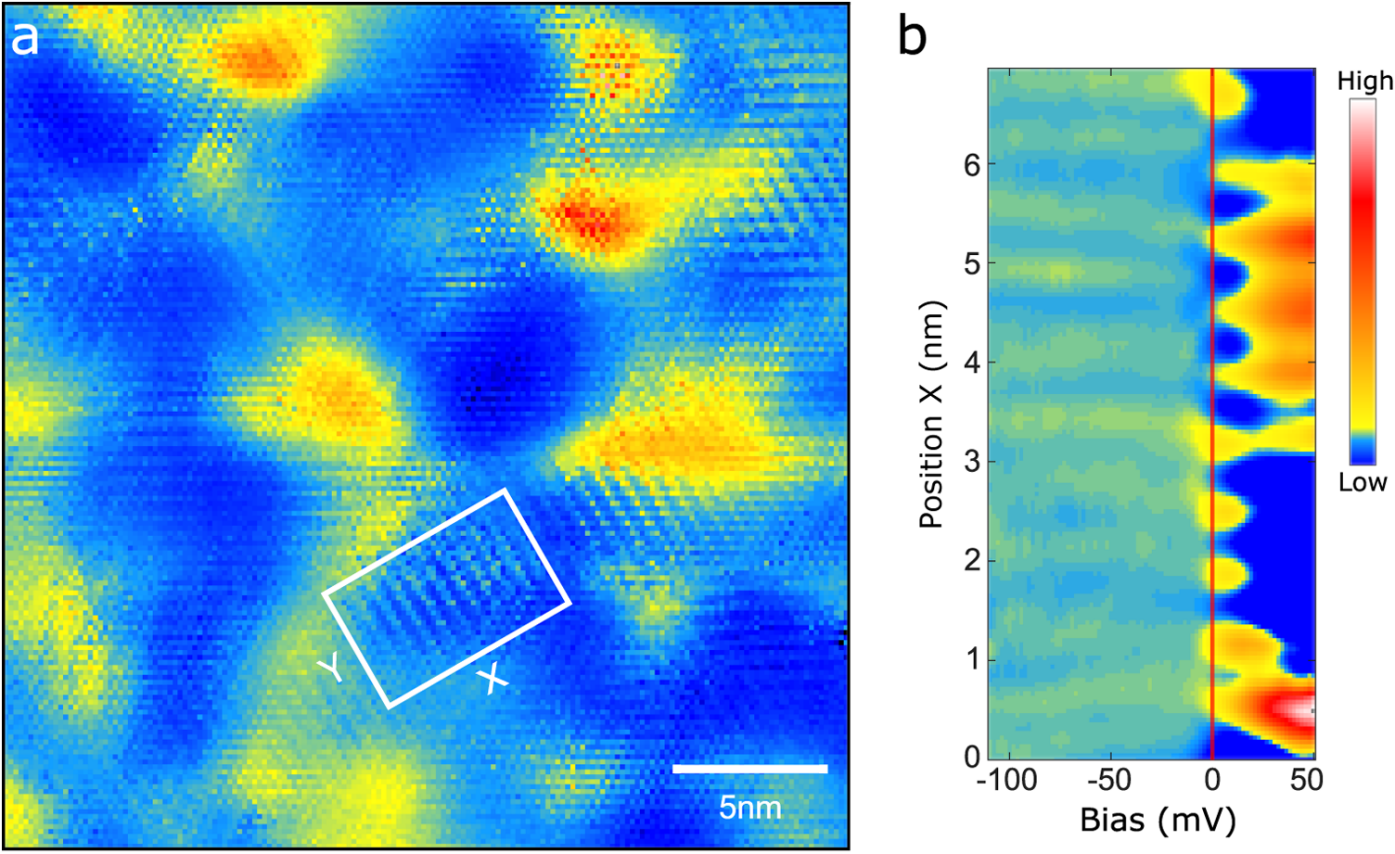
(a)  normalized STS map at −9 mV revealing CDW modulations. (b) *dI/dV*(*V*) map as a function of position and energy averaged along the *Y* direction at each position *X* over the white rectangle in (a), showing the CDW contrast inversion across *E*_*F*_.

Most relevant for the present study is that the CDW primarily develops in regions where local impurities induce electron doping. This is exemplified by the strong correlation between the dark defects seen at a large negative bias in Figure 1c and the blue regions corresponding to finite CDW amplitudes in Figure 1f. These dark defects correspond to electron doping by subsurface defects identified by blue stars in Figure 1f. The bright defects identified by arrows in Figure 1c,f correspond to local hole doping by other subsurface defects. Here, hole doping refers to the Fermi level locally shifting away from the conduction band (Figure S2). They shift the Fermi level below the conduction band edge, leading to suppression of the CDW amplitude. One may realize that the contrast in Figure 1b is opposite to the above defect analysis, with the CDW developing in the bright regions. This apparent contradiction is a direct consequence of the set point dependence of the tunneling current in the presence of a semiconducting gap. Indeed, for a large negative bias set point outside the semiconducting gap shown in Figure 3a, the integrated DOS available for tunneling is reduced as the Fermi level is shifting into the conduction band (i.e., for electron doping), and the tip will have to move closer to the surface to maintain a constant tunneling current. Hence, electron doping defects appear as depressions (i.e., dark in Figure 1c). The opposite is true when tunneling at a small negative set point voltage within the semiconducting gap (Figure 3b). In this case, the integrated DOS available for tunneling is larger when the Fermi level is shifted higher into the conduction band. Consequently, the tip has to be retracted to maintain a constant tunneling current and the corresponding surface region appears high (i.e., bright in Figure 1b).

**Figure 3:**
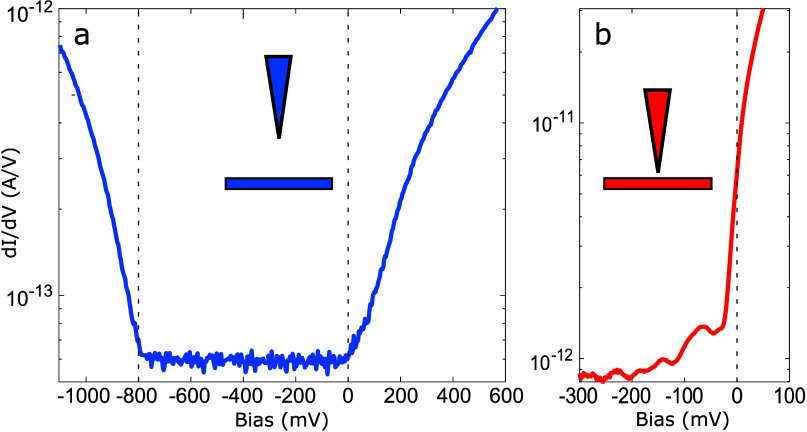
Two averaged *dI*/*dV*(*V*) spectra on a log scale from a grid with different set point bias, −300 and +600 mV for (a) and (b), respectively. The small inserts in each graph show conceptual sketches of the corresponding tip–sample distance.

The direct link between electron doping and the emergence of a CDW reconstruction is very explicit in Figure 4. Figure 4a shows the amplitude of the CDW Fourier component as a function of the position along the trace displayed in the inset. In Figure 4b, we present a grayscale plot of the tunneling conductance as a function of energy and position along the same trace. The minimum of the conduction band edge is at the Fermi level—highlighted by a red dashed line—in the regions devoid of any CDW modulations. Remarkably, we measure a finite CDW amplitude wherever the conduction band edge shifts below *E*_*F*_, with the appearance of a slight depression in the conductance near *E*_*F*_ corresponding to the CDW gap. To emphasize these characteristic spectral features, we consider the *dI*/*dV*(*V*) curves in three selected boxes outlined in Figure 4b and plot their average in the corresponding color in Figure 4c. The semiconducting gap extends to the Fermi level (yellow spectrum in Figure 4c) in the central region, where no CDW modulation is detected (yellow box). Conversely, in regions where a finite CDW amplitude is detected, the edge of the conductance band is significantly below *E*_*F*_, with a gap appearing at *E*_*F*_ (red and blue spectra in Figure 4c). Note that the gap is more pronounced in the region outlined in red, where the CDW Fourier component is stronger compared to that in the region outlined in blue.

**Figure 4:**
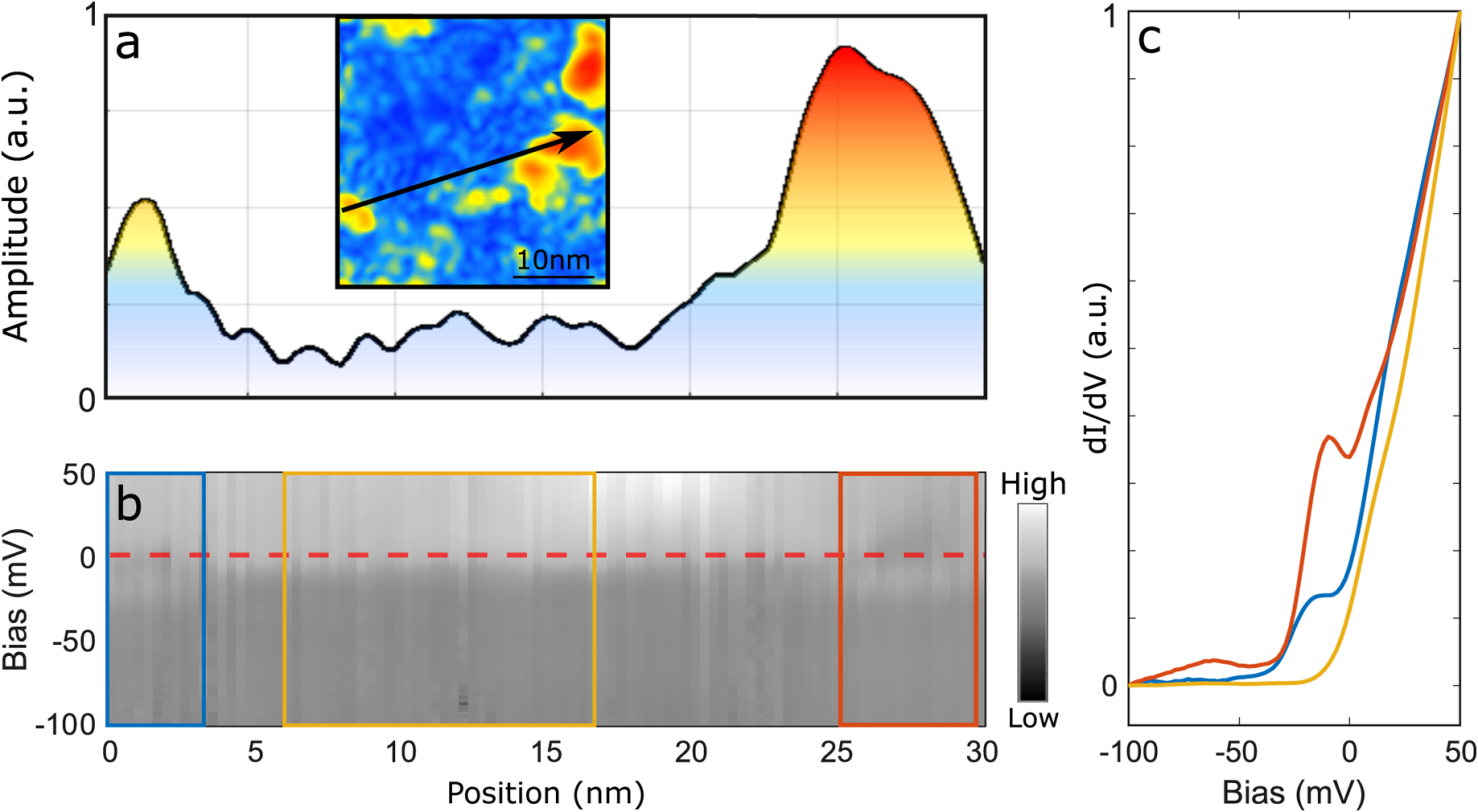
CDW modulation amplitude versus spectroscopic signal near *E*_*F*_. (a) CDW modulation amplitude along the black line in the inset CITS map. (b) Gray-scale plot of the *dI*/*dV*(*V*) spectra measured along the same line as panel (a). (c) Normalized *dI*/*dV*(*V*) spectra averaged over the boxes with corresponding color in panel (b).

Band structure calculations^37^ suggest that the CDW and QPI feature we observe on ZrSe_2_ originate in different electronic bands. The CDW lives in the lowest available conduction band, whose minimum lies at the *M*-point. The next band above *E*_*F*_ is a few hundred meV higher, with a minimum at the Γ-point. States at Γ (i.e., with small *k*_∥_) decay slower into the vacuum and thus contribute more to the tunneling current than states at the *M*-point with a large parallel momentum to the surface in a 2D crystal.^38^ Therefore, the bottom of the conduction band at the *M*-point can only be measured at low bias when the tip is close to the surface and when only the lowest energy states are sampled in the tunneling process. By increasing the tunneling bias, we probe deeper into the conduction band, resulting in the tunneling current being dominated by the states at the Γ-point, which are not involved in the CDW reconstruction but contribute to QPI. This explains the different nature of the periodic modulations resolved above and below *V*_bias_ = +100 meV.

Angle-resolved photoemission spectroscopy (ARPES) of alkali metal and copper intercalated ZrSe_2_^39−42^ further supports our data analysis. These experiments clearly show that electron doping from the intercalated atoms shifts *E*_*F*_ into the conduction band and forms electron pockets at the *M*-point. The *q*_1/2_ CDW modulation has been previously associated with Fermi surface nesting, connecting the pockets developing at the *M*-points.^20^ However, as the CDW contrast inversion tends to occur slightly below *E*_*F*_ (see Figure S1 for more examples of this contrast inversion), a phonon-softening mechanism is more fitting than the previously proposed FSN scenario. We did not introduce intentional dopant atoms into our crystals. However, stoichiometric ZrSe_2_ single crystals are challenging to grow and defects are native, as discussed above. The most likely defects in our crystals, whatever Zr interstitials or Zr-antisite occupation of Se vacancies they may be, are expected to electron dope the system. We find that the defect species that correlates strongest with the CDW modulation is only observable at high negative bias and is not visible in our atomically resolved topographies taken at lower positive bias. Chemical composition analysis suggests these defects are excess Zr intercalated in the VdW gap between the ZrSe_2_ layers, by analogy with the well-documented Ti intercalation in TiSe_2_. This causes us to categorize dark defects as subsurface electron doping defects.

In summary, analyzing scanning tunneling conductance maps as a function of energy, we find that intrinsic doping induces a 2*a* × 2*a* CDW modulation at the cleaved surface of bulk ZrSe_2_. Electron doping causes the Fermi level to shift into the conduction band, whose minimum sits at the *M*-point, which triggers the formation of a CDW reconstruction and the opening of a gap at the Fermi level. STS, ARPES, and theoretical band structure calculations explain the set-point-specific differences in spectroscopy and topography images in terms of momentum selectivity in the STM tunneling process. Our study unambiguously demonstrates the ability to tune the CDW phase transition by means of electron doping in a semiconducting bulk TMD. In addition to paving the way for applications exploiting tunable CDW ground states, these results enable further studies to understand the CDW formation mechanism. Of particular interest is quasiparticle interference imaging as a function of nonlocal doping using field effect or space charge doping to map the band structure in the vicinity of the Fermi level in the presence or absence of a CDW over the same area.

## Supporting Information Available

Additional contrast inversion maps, STS on electron and hole doping defects, strain analysis using geometrical phase analysis, and high-resolution STM topographies (PDF)
