# Supplementary material for: Doping Tunable CDW Phase Transition in Bulk 1T-ZrSe2
Source: Nano Lett. 2025 Jan 15;25(4):1729–35. doi: 10.1021/acs.nanolett.4c06377 (PMC11783592; doi:10.1021/acs.nanolett.4c06377)
Supplement: Supplementary file 1 — nl4c06377_si_001.pdf [file nl4c06377_si_001.pdf]

Supporting information

Doping Tunable CDW Phase Transition in Bulk 1T-$ZrSe_2$

Andreas Ørsted,* Alessandro Scarfato, Céline Barreteau, Enrico Giannini, and Christoph Renner*

University of Geneva, Department of Quantum Matter Physics, 24 Quai Ernest-Ansermet 1211 Geneva 4, Switzerland

E-mail: andreas.orsted@unige.ch; christoph.renner@unige.ch

Additional data showing CDW Contrast Inversion

In Figure S1, we provide additional collapsed constant current tunnelling spectroscopy (CITS) maps to show further examples of the CDW contrast inversion observed in our experiments. Figure S1(a) is a CITS map of $ZrSe_2$ with colored rectangles indicating the areas where the waterfall plots of Figures S1(b,c,d) are acquired. The waterfall plots are made by selecting a region and then summing the resulting dI/dV matrix along a direction of interest (one of the three lattice directions). The contrast inversion occurs slightly below E_F in all three examples illustrated in Figure S1(b,c,d).

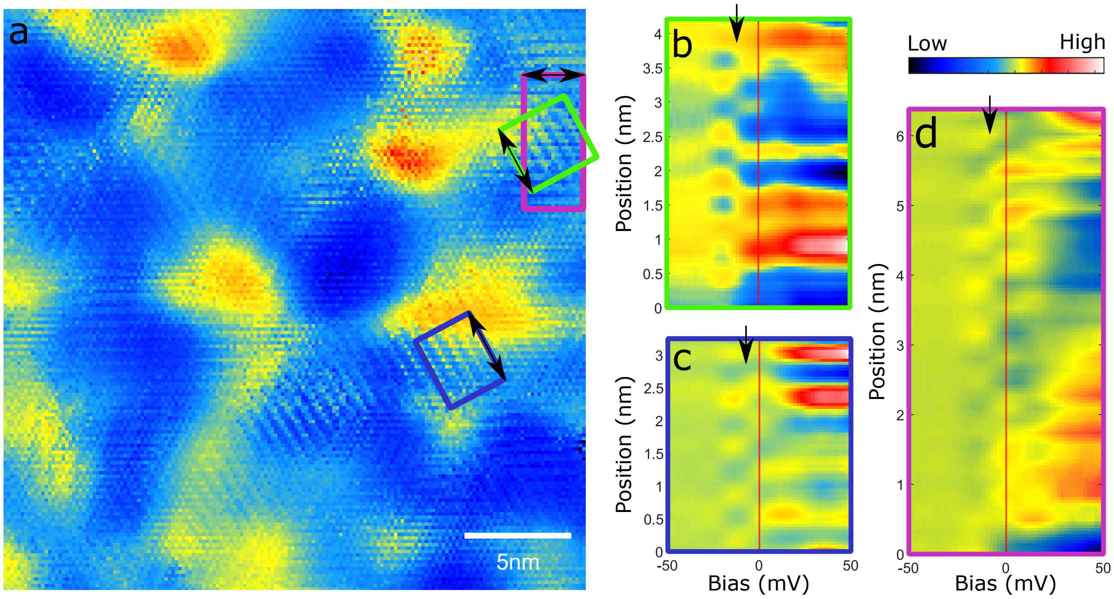

Figure S1. (a) $\frac{dI(V)/dV}{(I(V)/V)}$ normalized CITS map at −9 mV revealing CDW modulations. (b,c,d) CITS maps as a function of position and energy averaged along the directions marked by arrows in the corresponding colored rectangles in (a). The small black arrows in (b,c,d) highlight the CDW contrast inversion, which lies slightly below E_F.

Electron and Hole Doping From Dark and Bright Defect

The manuscript discusses how the dark and bright defects are doping electrons and holes, respectively. Figure S2(a) shows the same high negative bias topography as the one seen in Figure 1(c), revealing the subsurface defect positions. We highlight a dark and a bright defect by a blue and a red circle, respectively. Figure S2(b) shows the corresponding averaged STS measurement in the defect regions.

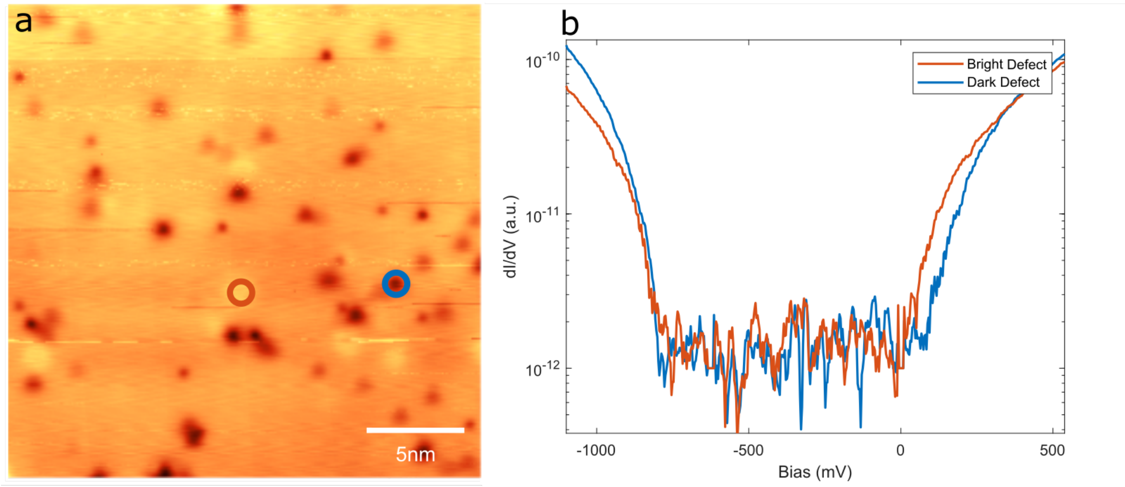

Figure S2. (a) STM topography of $ZrSe_2$ taken with -1100 mV bias with a bright and a dark defect marked by a red and a blue circle, respectively. (b) shows $\frac{dI}{dV}$ spectra taken at these positions with set-point bias of 600 mV.

The electron and hole doping nature of the defects results in sizeable shifts of the conduction band position in Figure S2(b).

Strain analysis

Figure S3 presents a comparison between the strain, evaluated by means of a Geometrical Phase Analysis algorithm,[1,2] with the spatial distribution of the CDW regions. The strain maps are extracted from the positive bias (V_b = +300 mV) topography in panel (a). (b) and (c) show the strain maps associated with the reciprocal space vectors indicated with a red and a green arrow in the Fourier transform in the inset of panel (a), respectively. To compare the strain with the spatial distribution of the CDW regions, in panels (e) and (f), the strain maps are superimposed to the negative bias topography in (d), where the CDW regions are clearly observed. It is clear that there is no correlation between strain and the distribution of CDW patches: the patches are not uniformly found in either high-strain or low-strain regions. The major part of the strain correlates with the atomic defect positions observed at positive bias.

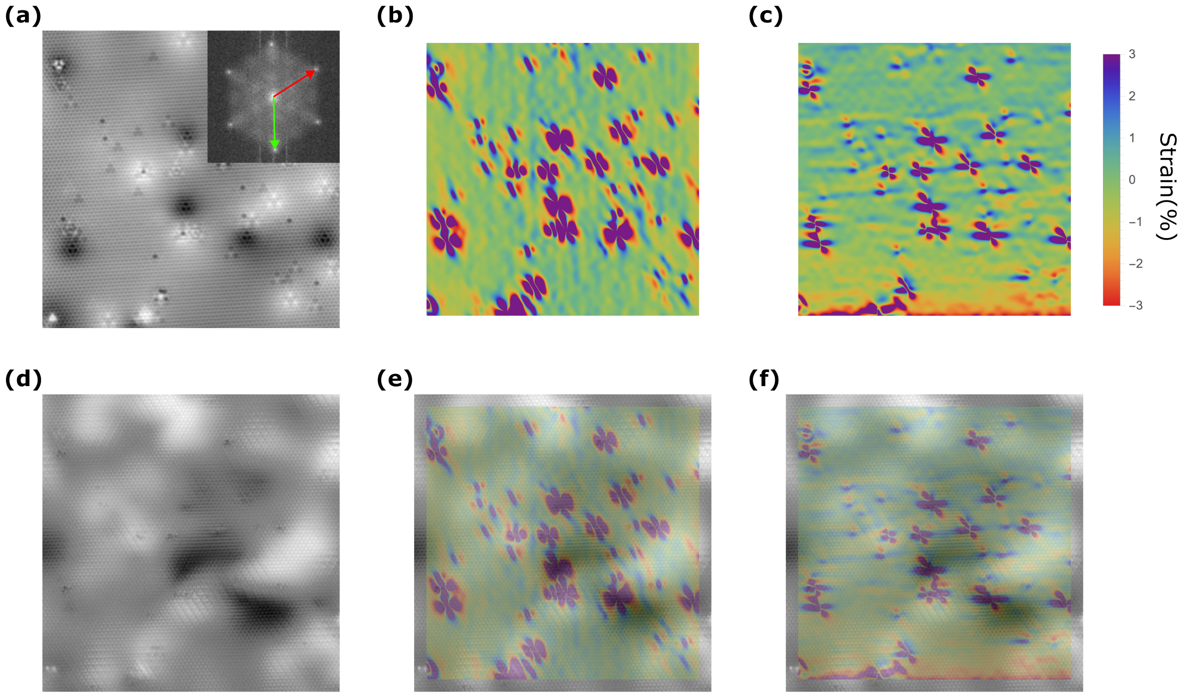

Figure S3. Strain analysis. The strain maps are extracted from the positive bias (V_b = +300 mV) topography in (a). In (b) and (c), the strain maps along the reciprocal space vectors are indicated with a red and a green arrow, respectively, in the Fourier transform in the inset of panel (a). The strain maps have been calculated by means of a Geometrical Phase Analysis algorithm.[1,2] In panels (e) and (f), the strain maps are superimposed to the negative bias topography in (d).

High-Resolution STM topography of $ZrSe_2$

Figure 1 of the original manuscript includes two atomically resolved STM topographies. However, we have added vector-based versions from the same area here to give the topographies their full potential. Note that the positive bias topography in Figure S4(a) shows a flat, perfect atomic lattice sprinkled with various atomic defects, whereas the negative bias topography in Figure S4(b) has a significant background and less resolved defects. The positive bias topography shows that the grooves observed at negative bias are of electronic origin.

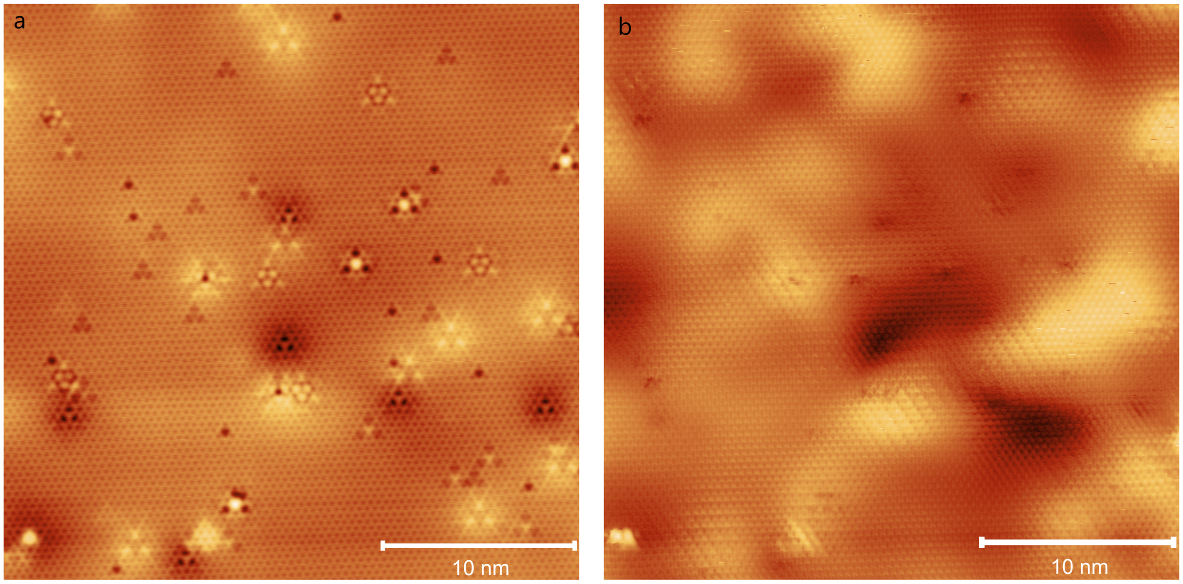

Figure S4. 30×30 nm^2 STM topographies of $ZrSe_2$ taken in the same area. (a) measured at +400 mV shows a flat atomic background and well-defined defects, whereas (b) measured at −400 mV shows a highly corrugated background with visible atoms but without well-defined defects.

References

(1) Lawler, M. J.; Fujita, K.; Lee, J.; Schmidt, A. R.; Kohsaka, Y.; Kim, C. K.; Eisaki, H.; Uchida, S.; Davis, J. C.; Sethna, J. P.; Kim, E.-A. Intra-unit-cell electronic nematicity of the high-Tc copper-oxide pseudogap states. *Nature* **2010**, *466*, 347–351.

(2) Fujita, K.; Hamidian, M. H.; Edkins, S. D.; Kim, C. K.; Kohsaka, Y.; Azuma, M.; Takano, M.; Takagi, H.; Eisaki, H.; Uchida, S.; Allais, A.; Lawler, M. J.; Kim, E.-A.; Sachdev, S.; Davis, J. C. S. Direct phase-sensitive identification of a *d*-form factor density wave in underdoped cuprates. *Proceedings of the National Academy of Sciences* **2014**, *111*, E3026–E3032.
